# A New TS Algorithm for Solving Low-Carbon Logistics Vehicle Routing Problem with Split Deliveries by Backpack—From a Green Operation Perspective

**DOI:** 10.3390/ijerph15050949

**Published:** 2018-05-10

**Authors:** Yangkun Xia, Zhuo Fu, Sang-Bing Tsai, Jiangtao Wang

**Affiliations:** 1 School of Traffic and Transportation Engineering, Central South University, Changsha 410075, China; jiaochyk166@163.com; 2 Zhongshan Institute, University of Electronic Science and Technology of China, Zhongshan 528400, China; jiangtao-w@foxmail.com

**Keywords:** vehicle routing problem, split deliveries, backpack, tabu search, low-carbon logistics, green economy

## Abstract

In order to promote the development of low-carbon logistics and economize logistics distribution costs, the vehicle routing problem with split deliveries by backpack is studied. With the help of the model of classical capacitated vehicle routing problem, in this study, a form of discrete split deliveries was designed in which the customer demand can be split only by backpack. A double-objective mathematical model and the corresponding adaptive tabu search (TS) algorithm were constructed for solving this problem. By embedding the adaptive penalty mechanism, and adopting the random neighborhood selection strategy and reinitialization principle, the global optimization ability of the new algorithm was enhanced. Comparisons with the results in the literature show the effectiveness of the proposed algorithm. The proposed method can save the costs of low-carbon logistics and reduce carbon emissions, which is conducive to the sustainable development of low-carbon logistics.

## 1. Introduction

With the deterioration of global climate, many countries have put forward the idea of low-carbon economy development. For instance, the Chinese government assumes that by 2020, the carbon emissions per unit of GDP will decline by 40–50% of that in 2005. In the manufacturing industry, food processing industry, express industry, and other industries closely related to human life, logistics operation is indispensable. Low-carbon logistics is the foundation of the sustainable development of the modern economy. Adopting some new green optimization technologies to reduce energy consumption, decrease carbon emissions, and save transportation costs is an important way of developing low-carbon logistics. For example, Maden et al. [1] designed a heuristic algorithm to solve the logistics vehicle routing problem (VRP) and found that their optimization technology can decrease carbon emissions by 7%. Therefore, the study of vehicle routing optimization is of great significance for promoting the development of low-carbon logistics and maintaining the sustainable development of green economy.

The costs of carbon emissions in transit can be measured indirectly through logistics distribution costs. In general, the fewer vehicles used and the shorter the travel time, the lower the cost of carbon emissions in transit. The existing VRP literature shows that using a strategy of split deliveries of the customer demand can save costs and facilitate the development of low-carbon logistics [2,3]. Dror et al. [2] proposed the vehicle routing problem with split deliveries (VRPSD) in 1989. The authors discussed the VRPSD and demonstrated that, compared with the non-split VRP (traditional VRP), the VRP with split deliveries can improve the vehicle loading rate, decrease the number of vehicles used, and shorten the travel distance. So, the method of split deliveries can also decrease carbon emissions. 

Subsequently, by integrating the vehicle load and customer demand as integers, Archetti et al. [3] constructed the classical VRPSD integer programming model (referred to herein as the K-VRPSD model) by assuming that the customer demand can be continuously split deliveries by unit, thus simplifying the solving of VRPSD. The previous VRP with split deliveries literature [3,4,5,6,7,8] basically followed the idea that the demand can be continuously split deliveries by unit, and works on the VRP with discrete split deliveries are relatively few. However, the discrete split form is widespread in practice. In the practice of logistics distribution, logistics enterprises often implement split packaging and delivery to save costs [8]. However, the type of goods, quality level, size, and other specifications are often different, which may not be suitable for one customer with one backpack. That is to say, each customer’s demand can be made up of multiple backpacks. In addition, enterprise often divides the orders of customer’s goods into several “backpacks” in e-commerce logistics, which are then delivered in batches. Once the backpacks of one customer are formed, it is not possible to split the customer’s demand any further. Compared with the classical customer demand non-split VRP, the demand of each customer in this paper can be a combination of multiple backpacks and customer demand can be split, but a single backpack cannot be split. That is to say, every customer’s demand can only be split deliveries by backpack. In this paper, backpack is defined as the smallest set of weight of customer demand that cannot be further split, and it is of significance to study the vehicle routing problem with split deliveries by backpack (VRPSDB). 

## 2. Literature Review

### 2.1. Low-Carbon Logistics

Many scholars have considered the importance of developing low-carbon logistics technology. Binford et al. [9] studied the regional logistics carbon budgets in carbon cycling research. By using some technical means—satellite remote-sensing methods coupled with micrometeorological and biomass measurements—they estimated the carbon exchange and storage rates. The low-carbon optimization technology has an important application value in many fields. Li et al. [10] proposed a novel three-stage network load profiling method to improve low voltage network visibility without extensive monitoring and to integrate low-carbon technologies in a cost-effective manner. In order to provide a better logistics service for the fashion apparel, Choi et al. [11] designed a new quick response (QR) system to enhance inventory management by reducing lead time. In addition to employing a faster delivery mode, their QR can also be achieved by local sourcing (instead of offshore sourcing). Choi et al. [11] also studied how the properly designed carbon footprint taxation scheme can be imposed on their QR system to enhance environmental sustainability via employing a local manufacturer by offsetting the probable higher total logistics and production costs. Lukman et al. [12] thought heavy-duty vehicles (HDVs) for winter services (e.g., snow plowing) were challenges to energy resources because HDVs are responsible for around 25% of CO_2_ emissions caused by road transportation. Based on a mathematical graph theory, they presented an optimization approach for the fleet management of winter services. From the calculated results, their optimization methods had a great effect on reducing carbon emissions and reducing logistics costs [12].

Some researchers also studied the low-carbon strategies in logistics operation in order to be compatible with governmental climate change policies to cut greenhouse gas emissions. Mckinnon [13] discussed the carbon emission limitation in the process of logistics distribution and put forward some green logistics operation strategies. He also deemed that freight modal split and a shift to lower carbon fuels can help the low-carbon development of the goods supply chain. Based on the evolutionary game theory, Gu et al. [14] added the low-carbon policy into the evolutionary game model between government and highway logistics enterprises. Combined with the calculation results of the model, Gu et al. [14] thought that many aspects can have impacts on the implementation of low-carbon strategies by government and highway logistics enterprises. They also pointed out that whether highway logistics enterprises implement low-carbon strategies is the consequence of the game between government and highway logistics enterprises and concerns a variety of issues. In order to help the e-commerce enterprise develop low-carbon logistics, Ji et al. [15] studied the strategies of low-carbon transport and service in e-commerce business. They established four decision models with different carbon confinement intensities and inferred the impact of more detailed carbon constraints on e-commerce delivery strategies. Perotti et al. [16] studied the strategies of green supply chain practices (GSCP) to improve company performance from a low-carbon logistics point of view. Melacini et al. [17] found that the search for synergies between traditional and online flows in both transport activities and warehouse is a key factor for economic and environmental sustainability. Their research is of great significance for promoting the sustainable development of enterprises and the construction of a green economy system [17]. 

### 2.2. Vehicle Routing Problem

The VRP has extensive application value in the low-carbon logistics distribution [18]. Toth et al. [18] extensively discussed the method and application of VRP. Kuo [19] pointed out that reducing carbon emissions has become an important issue, and that fuel consumption is also an important index in the VRP. Fuel consumption and carbon emissions are positively correlated with logistics distribution cost. Kuo [19] built a model of the time-dependent vehicle routing problem (TDVRP) for calculating the total fuel consumption cost. It is necessary to adopt some green optimization technologies to help the development of low-carbon logistics. Therefore, Kuo [19] designed a simulated annealing (SA) algorithm to solve the TDVRP. Through numerical experiments, he found that the proposed method provides a 24.61% improvement in fuel consumption cost. Through operational decisions—such as determining efficient vehicle routes and delivery schedules by considering time-varying traffic congestion in the service area—the logistics delivery enterprise can reduce the CO_2_ emissions [20]. Xiao et al. [20] studied a model of vehicle routing problem with CO_2_ emissions optimization and proposed a hybrid solution approach that combined a genetic algorithm with the exact dynamic programming procedure to solve the problem. Based on carbon tax policy in China, Wang et al. [21] constructed a green and low-carbon cold chain logistics distribution route optimization model with minimum cost and a cycle evolutionary genetic algorithm (CEGA) was used to solve the model. Their numerical experiments showed that the optimization model and algorithm can reduces costs and are conducive to the development of low-carbon logistics and green economy.

### 2.3. Vehicle Routing Problem with Split Deliveries

The vehicle routing problem with split deliveries (VRPSD) is a new research area of VRP. In the classical VRP, the customer’s demand cannot be split and delivered. However, in the VRPSD model, a customer’s order can usually be delivered in batches by multiple vehicles [2,3]. Archetti et al. [3] discussed the cost savings of VRPSD and showed that 50% in savings could be achieved under certain circumstances. Because customer demand can be split and delivered, VRPSD can usually reduce the number of vehicles used and shorten the travel time. So, we deem that the VRPSD can also reduce carbon emissions cost. Some scholars have discussed the optimization algorithms of VRPSD. For instance, Archetti et al. [3] used a tabu search (TS) algorithm. Aleman et al. [4] constructed a heuristic algorithm. Wilck-IV et al. [5] improved the genetic algorithm. Archetti et al. [6] proposed branch-and-cut algorithms. Rajappa et al. [7] designed an ant colony optimization and hybrid metaheuristics algorithm. Berbotto et al. [22] created a randomized granular tabu search heuristic algorithm. 

Some researchers also studied the expansion types of VRPSD. Ho et al. [23] used a TS algorithm for the VRPSD with soft time windows (VRPSDTW). Archetti et al. [24] designed a new branch and price-and-cut algorithm for the VRPSDTW. Belfiore et al. [25] proposed a scatter search (SS) approach for the fleet size and mix VRPSDTW (FSMVRPSDTW). Wang et al. [26] used a hybrid heuristic algorithm for the VRPSD with pickups and time windows (VRPSDPTW). Yan et al. [27] created a classic two-step solution algorithm for the multi-trip VRPSD with soft time windows (MVRPSDSTW). Nakao et al. [28] designed a dynamic programming based heuristic algorithm for VRP with discrete split deliveries (VRPDSD). Salani et al. [29] used a branch and price for the VRPDSD with time windows (VRPDSDTW). From the existing VRPSD literature [30,31,32,33,34,35], the study of VRP with discrete split deliveries (VRPDSD) is relatively rare, and the study of VRP with split deliveries by backpack (VRPSDB) is rarer. 

### 2.4. Comparison of Optimization Algorithms

The optimization algorithm can often help the logistics enterprises or the individual vehicle drivers make better vehicle scheduling with lower CO_2_ emissions and fuel consumption. Therefore, many experts have focused on the research of VRP optimization algorithm [1,2,3,4,5,6,7,8,9,10,11,12,13,14,15,16,17,18,19,20,21,22,23,24,25,26,27,28,29,30,31,32,33,34,35,36,37,38,39,40,41,42,43,44,45]. The VRP is an NP-hard problem [33]. An exact algorithm is difficult to use to solve large-scale VRP, so lots of scholars usually use a metaheuristic algorithm to solve it [1,2,3,4,5,6,7,8,9,10,11,12,13,14,15,16,17,18,19,20,21,22,23,24,25,26,27,28,29,30,31,32,33,34,35,36,37,38,39,40,41,42,43,44,45]. For example, the survey of metaheuristics for the VRP by Gendreau et al. [36] shows that a tabu search (TS) algorithm emerges as the most effective approach. Procedures based on pure genetic algorithms and on neural networks are clearly outperformed, while those based on simulated or deterministic annealing and on ant systems are not quite competitive. Fu et al. [37,38,39,40,41] designed a series of TS algorithms to solve the open VRP and closed VRP. They also compared and analyzed some other optimization algorithms, such as simulated annealing algorithm, genetic algorithm, ant colony optimization algorithm, particle swarm optimization algorithm, and so on. Through experiments, they point out that the TS algorithm has the advantages of simplicity, adaptability, and easy operation, and the TS algorithm usually performs better for solving large-scale VRP, especially when customer demand can be split and delivered [37,38,39,40,41]. Therefore, we designed a new TS algorithm to solve the VRP with split deliveries by backpack in this study. 

## 3. Mathematical Model

The capacitated vehicle routing problem (CVRP) is a basic type of the VRP (non-split VRP) [10]. Combining the classical CVRP, in this study, customer demand with the non-split condition was loosened to be split, but on the condition that it can only be split by backpack. Then, the capacitated vehicle routing problem with split deliveries by backpack (CVRPSDB) was constructed. The CVRPSDB refers to making a reasonable route for the vehicle with the lowest cost of low-carbon logistics distribution for the same type of vehicle, starting from the distribution center (vertex 0) to the customer *i* (vertex *i*). The vehicle must return to vertex 0 at the end, with some constraints: the capacity limitation of vehicles, split deliveries by backpack, and the limitation of route length (time). The distribution center is numbered as 0, and customers are numbered as 1, 2, …, *N*. The notations are defined as in Table 1.

The total costs of low-carbon logistics distribution (*F*) in transit can be measured indirectly through the number of vehicles used and the travel time. Therefore, we can define that *F* = *P*_1_*K* + *P*_2_*Z*. Here, *P*_1_ and *P*_2_ represent the coefficient of weight. In different transit environments, their values are different. When we calculate the value of *K* and *Z*, we only need to get the values of *P*_1_ and *P_2_* according to the specific transit environment, and then we can calculate the value of *F*. So, the key of logistics cost optimization is to calculate the optimal values of *K* and *Z*. Fu et al. [37] pointed out that the cost caused by the number of vehicles used is far greater than that of the cost of travel time; that is to say, *P*_1_ >> *P*_2_. In addition, the multiple objective optimization has become a hot spot in the field of modern logistics technology. Therefore, we will focus on logistics optimization technology and use a hierarchical approach to construct a double-objective mathematical model. The total low-carbon logistics distribution cost here contains two optimization objectives: the first is to minimize the number of vehicles used, the second is to minimize the travel time. The first one has priority. 

Assuming that the travel time between the vertexes coincides with the triangular inequality and the travel time represents the travel cost, the double-objective mathematical model of the CVRPSDB is as follows:

(1) min K

(2) $$\min\;Z=\sum_{i=0}^{N}\sum_{j=0}^{N}\sum_{k=1}^{K}\left(t_{ij}\cdot y_{ij}^{k}\right)$$

(3) $$\sum_{i=1}^{N}\sum_{r=1}^{R}\left(d_{i}^{r}\cdot x_{ir}^{k}\right)\leq Q,\;\;k=1,2,\ldots ,K$$

(4) $$\sum_{i=0}^{N}\sum_{j=0}^{N}\left(t_{ij}\cdot y_{ij}^{k}\right)\leq T,\;\;k=1,2,\ldots ,K$$

(5) $$\sum_{k=1}^{K}x_{ir}^{k}=1,\;\;i=1,2,\ldots ,N;r=1,2,\ldots ,R$$

(6) $$\sum_{k=1}^{K}\sum_{r=1}^{R}(d_{i}^{r}\cdot x_{ir}^{k})=d_{i},i=1,\ldots ,N$$

(7) $$\sum_{i=0}^{N}\sum_{k=1}^{K}y_{ij}^{k}\geq 1,\;\;j=1,2,\ldots ,N$$

(8) $$\sum_{i=0}^{N}y_{ie}^{k}=\sum_{j=0}^{N}y_{ej}^{k},\;\;e=1,2,\ldots ,N;k=1,2,\ldots ,K$$

(9) $$\sum_{k=1}^{K}\sum_{j=1}^{N}y_{0j}^{k}=K$$

(10) $$\sum_{k=1}^{K}\sum_{i=1}^{N}y_{i0}^{k}=K$$

(11) $$\sum_{i,j\in n\times n}y_{ij}^{k}\leq |n|-1,\;\;n=1,2,\ldots ,N;\;k=1,\ldots ,K$$

(12) $$y_{ij}^{k}\in \{0,1\},i,j=0,1,\ldots ,N;k=1,\ldots ,K$$

(13) $$K\geq K_{\min}=\left\lfloor \sum_{i=1}^{N}d_{i}/Q\right\rfloor \;+1$$

Equations (1) and (2) are objective functions; Equations (3) and (4) are the constraints for load and route length (time); Equation (5) limits that each backpack can only be delivered by one vehicle, that is, the customers’ single backpack cannot be split; Equation (6) is the condition for customer demand to split deliveries by backpack, that is, a customers’ demand is equal to the sum of its backpack demand, and every customer’s demand can be delivered by multiple vehicles with split deliveries by backpack, if needed; Equation (7) ensures that each customer is visited at least once; Equations (8)–(10) ensure that the intermediate customers have balanced vehicle flow, and all the vehicles return to the distribution center 0; Equation (11) eliminates constraints for the subloop to ensure that each vehicle visits a customer up to once; Equation (12) is an integer value constraint of 0 or 1; Equation (13) can estimate the lower bound of the number of vehicles used.

## 4. Design of Adaptive Tabu Search Algorithm

The VRP is an NP-hard problem [11], CVRPSDB is more complex than the classical VRP, and is also an NP-hard problem. It is difficult to effectively solve the large-scale NP-hard problems by using general exact algorithms [6]. Designing an intelligent heuristic algorithm is the general way to solve them. Fu et al. [37] pointed out that a TS algorithm is a preferable intelligent algorithm for solving VRP. In this work, an adaptive tabu search (ATS) algorithm was constructed for the CVRPSDB by adding adaptive operations and designing multi-neighborhood structures with reinitialization to enhance the global optimization ability.

### 4.1. The Expression of Solution and the Initial Solution

A solution is represented by using the arrangement between the vertex 0 and the customer backpack position, in which the vertex 0 can appear multiple times. A vehicle route can be formed by the closest two 0s with the backpacks in between. For example, in the solution $S=(0d_{1}^{1}d_{2}^{1}d_{2}^{2}d_{3}^{1}d_{3}^{2}d_{4}^{1}0d_{4}^{2}d_{4}^{3}d_{4}^{4}d_{5}^{1}d_{5}^{2}d_{6}^{1}d_{7}^{1}0\cdot \cdot \cdot 0)$, the first route is $\left(0d_{1}^{1}d_{2}^{1}d_{2}^{2}d_{3}^{1}d_{3}^{2}d_{4}^{1}0\right)$. We adopted the form of initial solution from Fu et al. [37], where the initial feasible solution was randomly generated. Specifically, we randomly generated the arrangement of customers from 1 to *N*. Then, under the vehicle load and work time constraints, followed by the backpacks of customer demand being added to the route one by one, a new vehicle route will be opened once there is a breach of the constraints. The initial solution is not split, that is, the same customer’s backpacks are all put into the same route.

### 4.2. Design of Multi-Neighborhood Structure

We designed a multi-neighborhood structure and used the random neighborhood transform strategy, randomly selecting a neighborhood for the current solution transform. Before the neighborhood operation, two different routes, $R_{1}$ and $R_{2}$, are randomly selected, and then a non-zero weight of customer or backpack in the two routes is randomly selected to carry out the transform. After each neighborhood operation, each of the adjacent 0s retains only the front-most one and merges the backpacks of the same customer within the same route. The neighborhood transform is randomly selected from the following five configurations. For example, in the solution $S=(0d_{1}^{1}d_{2}^{1}d_{2}^{2}d_{3}^{1}\underline{d_{3}^{2}}d_{4}^{1}0d_{4}^{2}d_{4}^{3}d_{4}^{4}d_{5}^{1}\underline{d_{5}^{2}}d_{6}^{1}d_{7}^{1}0\cdot \cdot \cdot 0)$ (the randomly selected backpacks $j_{1}$ ($d_{3}^{2}$) and $j_{2}$ ($d_{5}^{2}$) are underlined), the transform results are as follows:

- Insert in front of the vertex. Insert the vertex corresponding to backpack $j_{1}$ in front of the vertex corresponding to backpack $j_{2}$, and $S^{\prime }=(0d_{1}^{1}d_{2}^{1}d_{2}^{2}d_{4}^{1}0d_{4}^{2}d_{4}^{3}d_{4}^{4}d_{3}^{1}\underline{d_{3}^{2}}d_{5}^{1}\underline{d_{5}^{2}}d_{6}^{1}d_{7}^{1}0\cdot \cdot \cdot 0)$.
- Insert following the vertex. Insert the vertex corresponding to backpack $j_{1}$ following the vertex corresponding to backpack $j_{2}$, and $S^{\prime }=(0d_{1}^{1}d_{2}^{1}d_{2}^{2}d_{4}^{1}0d_{4}^{2}d_{4}^{3}d_{4}^{4}d_{5}^{1}\underline{d_{5}^{2}}d_{3}^{1}\underline{d_{3}^{2}}d_{6}^{1}d_{7}^{1}0\cdot \cdot \cdot 0)$.
- Vertex exchange. Exchange the vertexes corresponding to backpacks $j_{1}$ and $j_{2}$, that is, the backpacks corresponding to vertexes selected out of the two routes are all exchanged, and $S^{\prime }=(0d_{1}^{1}d_{2}^{1}d_{2}^{2}d_{5}^{1}\underline{d_{5}^{2}}d_{4}^{1}0d_{4}^{2}d_{4}^{3}d_{4}^{4}d_{3}^{1}\underline{d_{3}^{2}}d_{6}^{1}d_{7}^{1}0\cdot \cdot \cdot 0)$.
- Vertex exchange. Reverse the order of vertexes corresponding to backpacks $j_{1}$ and $j_{2}$, and $S^{\prime }=(0d_{1}^{1}d_{2}^{1}d_{2}^{2}\underline{d_{5}^{2}}d_{5}^{1}d_{4}^{4}d_{4}^{3}d_{4}^{2}0d_{4}^{1}\underline{d_{3}^{2}}d_{3}^{1}d_{6}^{1}d_{7}^{1}0\cdot \cdot \cdot 0)$.
- Backpack exchange. Exchange backpacks $j_{1}$ and $j_{2}$, and $S^{\prime }=(0d_{1}^{1}d_{2}^{1}d_{2}^{2}d_{3}^{1}\underline{d_{5}^{2}}d_{4}^{1}0d_{4}^{2}d_{4}^{3}d_{4}^{4}d_{5}^{1}\underline{d_{3}^{2}}d_{6}^{1}d_{7}^{1}0\cdot \cdot \cdot 0)$.

### 4.3. Evaluation of Solutions

In the process of search optimization, some infeasible solutions will help the algorithm to transit to a more feasible solution. In this paper, the number of candidate solutions generated from each iteration is p, where p=50+N, and candidate solution set A is constructed by deleting the $S_{candi}$ of $K<K_{\min}$. In order to guide the heuristic algorithm to solve large-scale VRP, ATS uses an adaptive penalty strategy in the search algorithm, setting the variable $F_{lag}$ to mark whether $S_{candi}$ is feasible. If feasible, $F_{lag}=1$ is marked, otherwise $F_{lag}=0$. According to previous experience from VRP-related literature [37,38,39,40,41], reduction in the number of vehicles has a greater effect on the shortening of the travel time and distance. The reduction in the number of vehicles is usually due to the increased vehicle loading rate and the avoidance of detour, and the feasible route is usually shorter (that is, the cost of time is usually less).

Setting two levels of evaluation indicators to select the new current solution $S_{now}$, the best solution $S_{best}$ (in which $S_{now}$ allows the violation of part of the constraints) needs to ensure the feasibility. In the first level of indicators, the number of vehicles K should be as few as possible; in the second level of indicators, the sum G of travel time costs Z and the penalty $Z^{\prime }$ for the violation of Equations (3) and (4) should be as small as possible. Values are as in the Equations (14) and (15), where $\delta_{k}$ represents the overrun of time spent on the *k*th route, and $\varepsilon_{k}$ represents the overload of the *k*th vehicle. After each iteration, the stratification is used to select the new $S_{now}$ from the candidate solution set A, and the non-tabu $S_{candi}$ with the minimum number of vehicles K is picked to form the solution set B (B⊆A), and then the solution with the minimum G value is selected from the set B. The new $S_{best}$ that is better than the original $S_{best}$ is selected from the feasible $S_{candi}$ (see details in Section 4.4).

(14) $$G=Z+Z^{\prime }$$

(15) $$Z^{\prime }=H\cdot [\alpha \cdot (\sum_{k=1}^{K}\delta_{k})+\beta \cdot (\sum_{k=1}^{K}\varepsilon_{k})]$$

α and β in Equation (15) represent the number of non-zero numbers in $\delta_{k}$ and $\varepsilon_{k}$, H is an adaptive penalty coefficient, value range H∈[20,2200], the initial value of H is taken as 100. If there are five iterations having non-feasible solutions in succession, they are multiplied by 2, and if there are five iterations having feasible solutions in succession, they are divided by 2. The setting of H can fully exploit the adaptive optimization potential of ATS and guide the algorithm to search alternately between feasible and non-feasible solutions and try to move closer to the feasible neighborhood.

### 4.4. Tabu Rules and Termination Conditions

The length of tabu has a great influence on the optimization performance of the TS algorithm. The larger tabu length can always accelerate the algorithm optimization, and the smaller tabu length can enhance the random diversity of the solution. In the early stage of the iteration, it is possible to avoid the repetition of search by increasing the length of tabu. In the middle and latter stages of iteration, the smaller tabu length can be used to enhance the richness of the neighborhood solution. In order to avoid cyclic search in the early search and enhance the random diversity of the middle and latter stages, the ATS sets the mixed tabu length by taking the fixed value 16 in the previous *m* iterations, and then takes the random integer from 5 to 16. Let m=500+15N.

Upon each candidate solution set A generated after each iteration, the current solution $S_{now}$ and the best solution $S_{best}$ corresponding to the next iteration is selected. Tabu list is an N×N matrix, and if a certain $S_{candi}$ is determined as $S_{now}$, then tabu operation is conducted for the neighborhood exchange vertexes i and j. That is, the matrix elements (i,j) in the tabu list are filled in with the corresponding tabu length. The length of the other tabu object minuses 1 after each iteration and is released when it is reduced to 0. The ATS also designed a tabu-breaking strategy; if a feasible $S_{candi}$ is better than the previous $S_{best}$ (that is, $S_{candi}\in A$ and $F_{lag}=1$, $K_{candi}<K_{best}$, or $F_{lag}=1$, $K_{candi}=K_{best}$, $G_{candi}<G_{best}$), then set the new $S_{now}$ and $S_{best}$, otherwise the best $S_{candi}$ ($S_{candi}\in B$) of non-tabu is set as the new $S_{now}$; if all $S_{candi}$ are tabued, then release the best $S_{candi}$ ($S_{candi}\in A$), and take the new $S_{now}$. In order to further avoid excessive tabu, after *m* steps of the iteration, reinitialization of the tabu list is carried out every *u* iterations, taking u=50.

There are two strategies to terminate the iteration, and either one can terminate. One is when the total number of iterations reaches the preset upper limit a; the second is when the iteration number of $S_{best}$, remaining unchanged, reaches the preset upper limit b. Take a=5000+150N, b=3000+5N.

### 4.5. Algorithm Description

The basic flow of ATS algorithm is described as follows:

Step 1: Initialize
Step 2: Input the relevant data and parameter values.
Step 3: Generate a feasible initial solution using a random strategy and take it as $S_{now}$ and $S_{best}$.
Step 4: **While** the termination condition is not met, **do**
Step 5: **While** the number of candidate solution is less than p, **do**
Step 6: Select a neighborhood in five kinds of neighborhood randomly by using random selection strategy.
Step 7: Transform $S_{now}$ by the selected neighborhood to generate a new candidate solution set A, and build the optimal non-tabu solution set B.
Step 8: **End**.
Step 9: Combine the previous analysis, if a feasible $S_{candi}$ is better than the original $S_{best}$, then set it as $S_{now}$ and $S_{best}$, otherwise the best non-tabu $S_{candi}$ ($S_{candi}\in B$) is set as the new $S_{now}$; if the set B=∅, release the best $S_{candi}$ ($S_{candi}\in A$), and take it as the new $S_{now}$.
Step 10: Update the tabu list.
Step 11: **End**.

## 5. Results and Discussion

### 5.1. Example Description

There are no benchmark problems yet for the VRP with split deliveries by backpack. Aleman used five CVRP large-scale examples (*N* ≥ 50, denoted as example a1, a2, a3, a4, a5) provided by Christofides in the study on the VRP with split deliveries by unit (belonging to the classical K-VRPSD) [4]. In this paper, the above examples are used to construct the examples for the VRP with split deliveries by backpack. Every customer’s demand is split into 1–4 backpacks randomly, and the route length (time) limit is added to construct the example. According to the characteristics of the original example, the Euclidean distance between the vertexes is used to represent the travel time, and the demand data of a4 with 150 customers is shown in Table 2.

### 5.2. Calculation Results

The ATS algorithm proposed was coded in Matlab2014a and implemented on a LENOVO^®^ V3000 laptop with CUP 2.40 GHz and 4 GB AMD. Each example was tested eight times, and the best result was taken.

The results show that the five examples have good performance. Besides the fact that example a5 needs 1 more vehicle, the rest are reduced to the least number of vehicles. The example a4 is used to illustrate the solution, with a *Z* value of 1063.80, where the customer 132 is dispatched by two vehicles with split deliveries by backpack, and the discrete split weights are 7 and 5. See Table 3 for details. 

### 5.3. Comparison Analysis

Aleman [4] used some heuristic algorithms to test the VRPSD examples from a1 to a5. They designed constructive approach (CA), iterative constructive approach (ICA), and iterative constructive approach plus variable neighborhood descent (ICA + VND). ICA + VND algorithm uses a two-stage solution, that is, the ICA results are taken as the initial solution of VND. See the details in literature [4]. The comparison results for *Z* value of CA, ICA, and ICA + VND, given by Aleman [4], are shown in Table 4.

In the number of vehicles used in the ATS, apart from example a5 needing 1 more vehicle, the other four examples all achieved the least number of vehicles used and have the same performance with the three contrasting algorithms. In travel time cost, the five examples of ATS are better than the comparative literature, and the saving ratio of CA, ICA, and ICA + VND was 3.66–10.34%, 3.30–8.40%, and 2.07–4.02%, respectively. Compared with the continuous unit split of Aleman [4], the ATS requires customer demand to be more stringent in terms of discrete split deliveries by backpack. From the size of the solution space, the solution set of the example in this paper should be a subset of the comparative example in the literature. That is, Aleman [4] should theoretically get better results than the ATS, but the actual results of the three algorithms are not as good as those of ATS. From the test results, the quality of the ATS solution is higher than that of CA, ICA, and ICA + VND, which indicates that ATS has a stronger search ability for optimization.

## 6. Conclusions

The VRP with split deliveries by backpack is a type of discrete split deliveries problem, which has wide application value in the practice of low-carbon logistics distribution. In this study, the VRP with split deliveries by backpack was studied, and a corresponding double-objective mathematical model was constructed. An adaptive tabu search algorithm with adaptive penalty mechanism, random neighborhood selection strategy, and reinitialization principle was designed. The test examples were constructed based on the benchmark problems. The computational results were compared with other relevant methods in the literature. Results showed that the model and its ATS algorithm can help us to find solutions with shorter travel time and lower costs of carbon emissions, and therefore help the development of low-carbon logistics and green economy. In future research, we can incorporate more constraints into the basic CVRPSDB model, and further improve the optimization performance of the ATS algorithm so that it can better serve the logistics practice. 

## Figures and Tables

**Table 1:** Notations of the capacitated vehicle routing problem with split deliveries by backpack CVRPSDB.

Symbols	Notations
*N*	The total number of customers.
*Z*	The total travel time for all vehicles.
*K*	The number of vehicles used (the number of routes).
*Q*	The vehicle load.
*T*	The maximum length of a route.
*d_i_*	The demand of customer *i*.
*d_i_^r^*	The demand of the *r*th backpack at customer *i*.
*R*	The maximum number in the number of actual backpacks of every customer.
*y_ij_^k^*	When the vehicle *k* directly goes to customer *j* from customer *i*, the value takes 1, otherwise 0.
*x_ir_^k^*	If the rth backpack at customer *i* is distributed by the vehicle *k*, the value takes 1, otherwise 0.
*t_i_*	The time when the vehicle reaches the customer *i*.
*t_ij_*	The direct travel time between the customer *i* and *j*.
*S*	A solution to the problem.
*S_initial_*	Initial solution.
*S_candi_*	Candidate solution.
*S_now_*	The current solution.
*S_best_*	The best solution.
*P*	The number of Candidate solutions.
*A*	Candidate solution set composed of *S_candi_*.
*a*	The upper limit of the total iteration number.
*b*	The upper limit of the iteration number of the “best solution” remaining unchanged.

**Table 2:** The customer demand data of a4.

Point No.	Demand	Backpack No.				Point No.	Demand	Backpack No.				Point No.	Demand	Backpack No.			
		1	2	3	4			1	2	3	4			1	2	3	4
1	10	2	2	6	0	51	10	1	3	3	3	101	7	2	3	2	0
2	7	1	3	3	0	52	9	4	5	0	0	102	30	14	12	4	0
…	…					…	…					…	…				
32	23	11	12	0	0	82	16	1	4	9	2	132	12	5	2	5	0
…	…					…	…					…	…				
49	30	2	4	12	12	99	9	1	2	1	5	149	18	1	2	5	10
50	13	6	7	0	0	100	17	3	3	9	2	150	10	3	6	1	0

**Table 3:** The results of a4.

Route	Travel Path	Route Length	Load Rate
1	0-13-117-97-92-59-95-94-112-0	42.25	72.00%
2	0-98-37-100-119-44-140-38-14-142-42-43-15-57-144-87-137-0	116.42	98.00%
3	0-52-106-7-123-19-47-124-48-82-0	78.68	75.50%
4	0-146-88-148-62-107-11-64-49-143-36-46-8-114-18-0	120.43	89.50%
5	0-96-93-85-91-141-86-16-61-104-99-6-0	74.35	95.00%
6	0-12-110-4-139-39-67-23-56-75-73-40-53-0	103.96	99.00%
7	0-76-77-3-79-129-78-34-135-35-136-65-66-128-20-122-**132(5+2)**-0	116.62	100.00%
8	0-138-1090-54-130-55-25-134-24-29-121-150-80-68-116-28-0	95.38	100.00%
9	0-27-69-101-70-30-131-32-90-63-126-108-10-31-127-0	83.27	95.00%
10	0-58-2-115-145-41-22-133-74-72-21-149-26-105-0	70.57	95.50%
11	0-89-118-60-83-125-45-17-113-84-5-147-0	77.84	98.50%
12	0-111-50-102-33-81-120-9-71-103-51-1-**132(5)**-0	84.03	99.50%

Note: The bold data 132 in the table is the split vertex number, with the corresponding split in parentheses.

**Table 4:** Comparison results of the adaptive tabu search (ATS) with others in the literature.

Pr.	*N*	*T*	*K*_min_	ATS	CA		ICA		ICA + VND	
				*Z*	*Z*	IMP/%	*Z*	IMP/%	*Z*	IMP/%
a1	50	180	5	**524.** **61**	578.83	10.34	568.67	8.40	540.82	3.09
a2	75	144	10	**846.22**	899.11	6.25	889.05	5.06	880.28	4.02
a3	100	207	8	**835.64**	873.46	4.53	863.18	3.30	854.13	2.21
a4	150	180	12	**1063.80**	1121.33	5.41	1108.97	4.25	1088.91	2.36
a5	199	180	16	**1362.33**	1412.18	3.66	1412.18	3.66	1390.55	2.07

Note: *Z* represents the distance value of the example; IMP represents the percentage of the comparative literature value *Z* (i.e., % greater than the ATS); the bold data represents the best value. CA: constructive approach; ICA: iterative constructive approach; ICA + VND: iterative constructive approach plus variable neighborhood descent.
